# Vascular clamping in liver surgery: physiology, indications and techniques

**DOI:** 10.1186/1750-1164-4-2

**Published:** 2010-03-26

**Authors:** Elie K Chouillard, Andrew A Gumbs, Daniel Cherqui

**Affiliations:** 1 Department of Surgery, Centre Hospitalier Intercommunal, Poissy, France; 2 Department of Surgical Oncology, Fox Chase Cancer Center, Philadelphia, PA 19111, USA; 3 Digestive Surgery Department, Hôpital Henri Mondor - University Paris-XII, Créteil, France

## Abstract

This article reviews the historical evolution of hepatic vascular clamping and their indications. The anatomic basis for partial and complete vascular clamping will be discussed, as will the rationales of continuous and intermittent vascular clamping.

Specific techniques discussed and described include inflow clamping (Pringle maneuver, extra-hepatic selective clamping and intraglissonian clamping) and outflow clamping (total vascular exclusion, hepatic vascular exclusion with preservation of caval flow). The fundamental role of a low Central Venous Pressure during open and laparoscopic hepatectomy is described, as is the difference in their intra-operative measurements. The biological basis for ischemic preconditioning will be elucidated. Although the potential dangers of vascular clamping and the development of modern coagulation devices question the need for systemic clamping; the pre-operative factors and unforseen intra-operative events that mandate the use of hepatic vascular clamping will be highlighted.

## Introduction

Efforts to reduce or eliminate operative bleeding, have been the primary focus throughout the history of liver surgery. For years the degree of hemorrhage has remained a major prognostic factor after liver resection. Vascular clamping is an efficient tool to minimize bleeding during parenchymal transection. This has been made possible by the liver's known tolerance to normothermic ischemia. Different types of clamping methods have been described including total (i.e. Pringle maneuver) and partial or selective (i.e. selective clamping of the part of the liver to be resected) **(APPENDIX 1)**. In addition, clamping can be applied to the inflow only, or to both inflow and outflow (hepatic vascular exclusion). Clamping may also be either continuous or intermittent.

The indication, as well as the type of clamping, depends mainly on the size and the location of the lesions to be resected, the quality of the liver parenchyma, the surgeon's preferences, and the unexpected operative events. Ideally, the type of clamping is decided preoperatively. Operative hemodynamic and fluid management differs according to the type of clamping. For example, in the absence of inferior vena cava clamping, fluid expansion must be limited while such an expansion is required with caval clamping. Therefore, close collaboration between surgeons and anesthesiologists is required to achieve a safe liver resection.

## Anatomic and Physiologic Basis of Liver Vascular Clamping

### Hepatic Inflow

The adult liver, the largest organ in the body, accounts for 2% to 3% of the overall body weight. Richly vascularized, the liver has an inflow carried through the portal vein and the hepatic artery and an outflow draining through the main and accessory hepatic veins. The main portal vein drains the splanchnic territory and carries 70% to 80% of overall hepatic inflow. It divides into two branches, the right and the left portal veins, which divide into sectoral and segmental branches. Portal clamping may be applied to the main portal vein or to one of its lobar or more distal branches. Blood pressure in the main portal vein is usually low with a portocaval gradient (i.e., portal vein pressure minus central venous pressure) of less than 5 mmHg. In chronic liver disease, especially cirrhosis, the portocaval gradient may be increased to the point of portal hypertension (i.e. portocaval gradient >10 mmHg).

The hepatic artery supplies 20 to 30% of the liver inflow and 50% of its oxygen supply. It divides, identically to the portal system, into lobar, sectoral and segmental branches, and clamping can be applied to the hepatic arterial trunk or to its more distal branches. Blood pressure is, of course, much higher in the hepatic arteries as compared to the portal system. In the most common anatomy, the main hepatic artery arises from the celiac trunk. In 20 to 25% of cases, several types of anatomic variations may be encountered. The most common ones include the right hepatic artery arising from the superior mesenteric artery and running behind the pancreatic head along the right posterolateral flank of the portal vein, and the left hepatic artery arising from the left gastric artery and running in the lesser omentum. The proper identification of these vessels is mandatory if complete and effective clamping is to be achieved. Inflow vessels, either portal or arterial, run and bifurcate together alongside a corresponding bile duct, starting in the *porta hepatis *and then into the liver through the hilum surrounded by a glissonian sheath. Inflow vessels may be clamped together, without prior dissection and with bile ducts, or separately after being dissected and encircled.

The regulation of arterial flow occurs through an arterial adenosine-dependant humoral paracrine pathway. In order to maintain a relatively stable overall hepatic inflow, arterial vasodilation occurs in cases of decreased portal flow and vasoconstriction in cases of increased portal flow. When portal inflow decreases, adenosine concentration increases, resulting in arterial vasodilation [1]. The opposite occurs if portal inflow increases. Portal inflow itself is not regulated but depends on the splanchnic (mesenteric) flow and the hepatic resistance. In cases of decreased portal inflow due to intrahepatic block, portal thrombosis or portacaval shunt, hepatic arterialization of the liver occurs. In cases of hypervascularized tumors, the hepatic artery may be unusually large.

### Hepatic Outflow

In the hepatic sinusoid, arterial and portal blood flows mix and drain into the centrolobular veins, which drain into venules, which eventually form the hepatic veins.

There are three major hepatic veins (right, middle, and left), all draining into the termination of the abdominal inferior vena cava (IVC) near the diaphragm and the right atrium. This anatomic location near the thoracic low-pressure zone is crucial to maintain an optimal venous drainage of the liver. The 15 to 20-mm diameter main hepatic veins have a very short extrahepatic segment. The left and the middle veins usually join together to form a common trunk just before entering the IVC. In 10% of cases, a large accessory right inferior hepatic vein is present, mainly draining segment 6. The existence of such an accessory vein should be identified upon preoperative imaging, using operative ultrasound or during caval dissection. Segment 1 and 9 drain through 1 to 3- mm diameter spigelian veins directly into the IVC.

Blood pressure in the hepatic sinusoids depends on hepatic blood flow, hepatic resistance, and pressure in the hepatic veins; the latter is directly linked to the pressure in the IVC and right atrium and, therefore, can be estimated by monitoring of the central venous pressure (CVP) during open procedures. In cases of inflow clamping, intrahepatic blood pressure as well as pressure in the hepatic veins depends exclusively on CVP.

Unfortunately, during laparoscopy the pneumoperitoneum renders CVP readings unreliable.

In these cases, CVP must be estimated by observing the IVC. When the IVC appears flaccid and fluctuates with movements of the heart and lung it can be assumed that the CVP is low and suitable for parenchymal transection. Hepatic outflow clamping may be obtained by caval clamping or by direct clamping of the main hepatic veins.

## VASCULAR CLAMPING TECHNIQUES: Inflow Occlusion

### Pringle Maneuver

J. Hogarth Pringle, in 1908, described the efficacy of hepatoduodenal ligament clamping in cases of liver trauma. It is the simplest and the most widely used method of liver vascular clamping [2]. The hepatoduodenal ligament is usually clamped *en masse*. Once the lesser omentum is opened, a blunt dissector may be passed through the foramen of Winslow and the hepatoduodenal ligament encircled with an umbilical tape.

When a right hepatic artery originating from the superior mesenteric artery is present, it runs within the hepatoduodenal ligament and is therefore included in the Pringle maneuver. By contrast, a left hepatic artery originating from the left gastric artery runs separately and requires individual clamping in order to totally occlude the arterial inflow. Individual vascular structures of the hepatoduodenal ligament can be identified separately and clamped. This method is used when dissection of the hepatoduodenal ligament is performed prior to resection, as in cases of hilar tumors where hepatoduodenal ligament dissection is indicated.

*En masse *Pringle maneuver can be performed using atraumatic flexible clamps or a tourniquet. In cases where separate clamping of portal and arterial vessels are used, small atraumatic vascular clamps or Rumel tourniquets are used.

### Selective Clamping: Major Hepatectomy, Sectorial, or Segmental

This method consists of selective clamping of a hepatic lobe, a section, or even a liver segment. The aim is double: (1) to limit the ischemic injury to the projected resection and (2) to accurately demarcate anatomic territories by creating ischemic margins at the liver surface. For major hepatectomy, the surgeon can devascularize the corresponding liver lobe by dividing the unilateral portal pedicle. Parenchymal transection can then be performed along the line of demarcation of the ischemic area. Another method consists in clamping the unilateral pedicle and division of it more distally during parenchymal section.

Selective sectoral clamping is mainly used for right liver sections (i.e., anterior section including segments 5 and 8 or posterior section including segments 6 and 7). It identifies anatomic resection margins in some complex, segment-based hepatectomies, including central (segments 4, 5 and 8), right anterior (segments 5 and 8) and right posterior (segments 6 and 7) liver resections [3]. Two approaches may be used in order to control lobar and sectorial pedicles: the intraglissonian dissection and the suprahilar approach.

### Intraglissonian Dissection and Clamping

This technique is based on an extensive dissection of the hepatoduodenal ligament. To approach the right pedicles, the right posterolateral peritoneal fold of the hepatoduodenal ligament is opened behind the common bile duct, allowing for identification and dissection of the main portal vein. Dissection is then pursued cephalad towards the portal bifurcation in order to control the right portal vein. The 1-cm long right portal branch can be carefully encircled. Dissection may be pursued distally in order to control the anterior and posterior sectoral portal branches. The same type of dissection is used for arterial branches. When anatomy is modal, the right branch of the hepatic artery is located behind the common bile duct and in front of the portal vein. Arterial sectorial branches can then be dissected and controlled. When an additional right hepatic artery exists, it is located along the right posterolateral flank of the portal vein. The existence of such an artery does not exclude the possibility of coexistence of a right branch of a hepatic artery arising from the celiac trunk.

Dissection on the left side of the portal pedicle is usually easier. The left branch of the hepatic artery runs on the surface of the upper left side of the hepatic pedicle. After incision of the peritoneal fold of the hepatoduodenal ligament, the left arterial branch is easily identified and controlled. The left portal vein is located just behind its corresponding artery and is easily identified along its long extrahepatic segment, also known as the *pars transversus*. It gives rise to two or three spiegelian veins going to segment 1. Again, looking for and controlling an additional left hepatic artery must be performed.

### Suprahilar Dissection and Clamping

Suprahilar dissection consists of controlling and clamping *en masse *the lobar and sectorial pedicles with their glissonian covering as in Pringle maneuver[4,5]. However, in contrast to Pringle maneuver, suprahilar dissection of more distal pedicles is sometimes more difficult to achieve. First, Glisson's capsule is opened just above the liver hilum in order to mobilize the hilar plate. A second incision in Glisson's capsule is performed behind the liver hilum, in front of the caudate lobe, just to the left of the area where the right portal pedicle penetrates into the liver. A blunt right angle dissector may then be inserted around the liver hilum in order to encircle it, using umbilical tapes. The surgeon must widely open Glisson's capsule, stay in the vicinity of the hilar plate identified by its whitish color, and finally avoid forceful dissection. The distance separating the two incisions is always longer than expected. Although relatively common, bleeding during this maneuver, usually of venous origin, is rarely massive and usually stops spontaneously after the passage of the tapes. The application of a hemostatic sponge may also be helpful. Once the suprahilar umbilical tape is in place, it may be oriented to one side or another in order to perform unilateral clamping of the liver to be resected [6].

Sectoral pedicles may be dissected according to the same principles since their origin is extrahepatic. Usually, dissection starts with suprahilar control of the right portal pedicle. When pulled down, it facilitates the identification of the sectoral pedicles. An additional incision in Glisson's capsule is performed at the level of the sectoral pedicle followed by its encircling by an umbilical tape using a dissector against the whitish glissonian sheath. Selective anterior or posterior right sectoral clamping may then be performed. In the suprahilar approach, clamping is better performed by a tourniquet.

### Segmental Clamping

This technique was first described by Shimamura et al. and then developed by Castaing et al. [7,8]. It consists of selective clamping of a segmental or a subsegmental portal branch using a vascular balloon associated with unilateral clamping of the lobar arterial branch. The portal branch is identified by peroperative ultrasound and punctured with a fine needle in order to introduce a floppy wire to serve as a leader to a dilator. Finally, a balloon-equipped catheter is inserted in the venous lumen in order to occlude the portal branch. The arterial branch is dissected and then clamped at the level of the hepatic pedicle. Inside the catheter, a channel allows the injection of methylene blue dye beyond the balloon into the occluded vein in order to delineate the devascularized liver. The blue-dyed liver can then be resected. The aim of the technique is to perform an anatomic resection of the segment containing the tumor. Although elegant, this technique is seldom used.

Intrahepatic vascular division characterized the first technique of liver resection, consisting of clamping "en masse" of the hepatic pedicle and then sectioning the parenchyma according to the anatomical lines with intraparenchymal dissection and ligation of the glissonian pedicles [9]. Subsequently, a French group described an extrahepatic division of the corresponding glissonian pedicle (i.e., portal and arterial branches) prior to the performance of the parenchymal transection [10,11].

A third technique, combines the former two, and consists of extrahepatic control and clamping of the arterial and portal vessels while vascular division is performed intrahepatically after parenchymal transection [12,13]. The advantage of extrahepatic dissection lies in the anatomic delineation of the projected liver resection while that of intraparenchymal division avoids unnecessary parenchymal devascularization and necrosis. In the two latter methods, devascularization affects only the liver segments to be resected, allowing resection to be carried out with or without clamping of the remaining liver vessels. However, in cases of hemorrhage, the Pringle maneuver may be necessary.

According to one of the largest European experiences, unilateral clamping may be safely used in up to 80% of major hepatectomies for peripherally located liver tumors. Total clamping is only used in the remaining cases due to uncontrolled bleeding [14].

#### The Role of Central Venous Pressure

Continuous collaboration between the anesthesiologist and the surgeon is a required for the success of a liver resection. The principle is even more important in achieving low central venous pressure (CVP), which currently represents the gold standard in minimizing blood loss during parenchymal transection. When inflow clamping is used, the hepatic veins remain patent and bleeding may continue via these vessels. The pressure within the liver from the hepatic veins to sinusoids is directly related to the pressure in the IVC, which is directly dependant on the CVP. The hypothesis that low CVP would be accompanied by a low pressure in the hepatic veins and thereby decreasing blood loss during transection has been tested in several studies after having been evoked in clinical practice for the first time in the early nineties [15].

It is probable that a reduction in blood loss and blood transfusion requirements can be achieved if the CVP is maintained below 5 cm H2O during parenchymal transection. Such a reduction does not seem to be influenced by the type of inflow clamping, the resection type, the length of the procedure, the type of liver disease, or the method of liver transection [16]. The potential for air embolism while maintaining a low CVP may be significant. A negative CVP can allow large volumes of air through small or unrecognized lacerations of the hepatic veins. This is unlikely with a positive CVP. Immediate control of significant holes in the hepatic veins must be undertaken. End-tidal carbon dioxide must be carefully monitored. When low CVP cannot be achieved, clamping of the infrahepatic IVC has been utilized [17,18].

#### Vascular Clamping During Laparoscopic Liver Resection

The same principles of vascular clamping are applied in laparoscopic liver surgery. Inflow control, either total or unilateral, is the most commonly used technique.

Pneumoperitoneum generates some degree of counter pressure, which may reduce bleeding. Despite low CVP during surgery, the theoretical risk of gas embolus has not been confirmed in reported series, CO2 being soluble [19,20]. However, caution still applies.

During laparoscopy, clamping is possible and well tolerated [21]. The Pringle maneuver is used most commonly in patients with a diseased liver, in patients with major resections, or when intra-operative hemorrhage occurs. Extrahepatic control of the hepatic veins can be used, either for the left and middle hepatic veins, or for the right hepatic vein [22-24].

## VASCULAR CLAMPING TECHNIQUES: Outflow Occlusion

This type of clamping includes clamping of the IVC above and below the liver. It may be more selective by clamping the hepatic veins themselves, leaving the caval flow undisturbed. Outflow clamping should always be preceded by inflow occlusion.

### Total Vascular Exclusion of the Liver (TVE)

Total vascular exclusion (TVE), the most complete liver clamping method, entails exclusion of the liver from the splanchnic and systemic circulations by total inflow occlusion associated with clamping of the IVC below and above the liver [25-28]. Afferent clamping consists of total clamping of the hepatic pedicle, usually massively.

Identifiction and clamping of accessory arteries is mandatory because an incomplete afferent clamping associated with an efferent clamping leads to liver congestion and hemorrhage. A straight clamp inserted from the right side to the left side is most commonly used for the sub-hepatic IVC while a Satinsky clamp inserted from the left to the right is the most appropriate for the suprahepatic IVC. Caval clamping necessitates complete hepatic mobilization after division of the hepatic ligaments. Both the infra and supra-hepatic IVC are dissected and encircled. The retrohepatic IVC must be liberated from the posterior plane. It is essential to dissect and divide each non-hepatic branch in order to annihilate the possibility of blood reflux in the excluded caval segment. Usually, the adrenal vein is the only non-hepatic, retrocaval, afferent branch. This vein must be ligated and divided. Although infrahepatic caval clamping may be applied above the level of the adrenal vein, the division of this vein is the most appropriate and sure method of clamping. The phrenic veins (there are usually 3) draining into the IVC at the level of the main hepatic veins, must also be systematically clamped. This can usually be achieved through the suprahepatic caval clamping.

In some patients with tumors of the hepatic dome, suprahepatic caval clamping is not possible through standard laparotomy. A thoraco-abdominal incision is usually required for total IVC clamping. This is mainly done from the right to the left. Occasionally, in order to achieve TVE, the surgeon needs to perform intrapericardial caval clamping. In these cases, the phrenic veins are not included in the clamping and TVE is rather incomplete. It may then be necessary to ligate the phrenic veins. A well-achieved TVE allows for a bloodless operating field during hepatic parenchymal transection.

Liver congestion or persistant bleeding during transection indicates incomplete TVE. When this occurs, the completeness of pedicle or caval clamping must be checked (can occur with insufficient clamp occlusion or inadequate clamp application) or an accessory artery or a non-hepatic, non-ligated vein pouring into the excluded caval segment should be sought (i.e., adrenal vein, phrenic vein). The following order in clamp application should be performed: hepatoduodenal ligament, infrahepatic IVC, and suprahepatic IVC. During liver resection under TVE, hilar dissection prior to transection is optional. Some authors avoid it completely and divide the inflow within the parenchyma at the time of transection29. Others still perform hilar dissection and extrahepatic inflow division, thus reducing the clamping time [29]. The liver parenchyma is transected according to the general principles of transection.

After the resection is completed, the suprahepatic clamp is released; any major bleeding is controlled with suture ligatures before removing the remaining clamps. The infrahepatic IVC and portal clamps are removed, and any further bleeding is controlled. When TVE can be tolerated, it ensures the best way to minimize intra-operative blood loss **(APPENDIX 2)**. However, it is associated with profound volume shifts, which can severely complicate the post-operative course. Venovenous bypass or vascular exclusion preserving the caval flow can reduce these complications, but adds to the time and complexity of the procedure [16,30].

### Hepatic Vascular Exclusion with Preservation of the Caval Flow (HVEPC)

With this method, caval clamping is replaced by hepatic vein clamping. When compared to TVE, HVEPC has three advantages: (1) improved hemodynamic tolerance (2) it allows intermittent clamping; and (3) it can be used to perform selective exclusion of a part of the liver. However, HVEPC necessitates a wider dissection of the liver with extrahepatic control of the hepatic veins. Total HVEPC consists in clamping the afferent pedicles as well as the right, the middle and the left hepatic veins. HVEPC is not really total unless segment 1 is completely disconnected from the IVC by division of the spigelian veins. Otherwise, the liver remains included in the systemic circulation by the means of caudate lobe veins.

Left partial HVEPC excludes the left liver (segments 2 to 4) and part of the right anterior sector (segments 5 and 8); it involves clamping the *porta hepatis *(and not the left portal pedicle), the left hepatic artery if present, and the left and middle hepatic veins.

Right posterior partial HVEPC excludes the right posterior section (segments 6 and 7) and involves clamping the *porta hepatis *or the right portal pedicle (portal and arterial branches to the right lobe) and the right hepatic vein, plus clamping or division of the right inferior hepatic vein if present. Right partial HVEPC, excluding the right lobe (segments 5 to 8), is feasible only in cases where the left and middle hepatic veins have separate caval insertion; it involves clamping the *porta hepatis *and the right and the middle hepatic veins.

To perform HVEPC, the falciform ligament is completely divided to expose the suprahepatic IVC and the confluence of the major hepatic veins. The right hepatic vein is controlled extrahepatically. After complete mobilization of the right lobe of the liver, the right and anterior aspects of the IVC are dissected by division of minor hepatic veins and division of the right aspect of the retrocaval ligament, progressing cranially, until the right hepatic vein is exposed and encircled. When a right inferior hepatic vein is present, it is either encircled or ligated and divided according to its size and the side of the resection. When the hepatectomy includes the resection of segment 1, the latter is completely separated from the IVC by division of all of its venous branches until the liver is connected to the IVC by the three major hepatic veins only. The control of the left and the middle hepatic veins starts by mobilization of the left hepatic lobe and division of the lesser omentum. The junction of the left hepatic vein and the IVC is then exposed by division of the peritoneal reflection above the caudate lobe, with or without division of the *ligamentum venosum*. In most cases, the left and middle hepatic veins form a common trunk and are encircled together, usually from left to right. In some cases, the left and middle hepatic veins are looped separately. The rest of the procedure is conducted as in liver resection under TVE.

HVEPC permits a bloodless resection in almost all cases of continuous clamping. In rare cases, some venous bleeding occurs due to the non-separation of segment 1 from the IVC, especially when the CVP is high. Whereas conventional selective inflow clamping is applied according to the anatomy of the portal pedicles, partial HVEPC is based on hepatic venous anatomic territories. The left hepatic liver is drained by the middle and left hepatic veins, which commonly form a common trunk. Clamping this common trunk is sufficient to perform formal left hepatectomies, and clamping the right hepatic veins is unnecessary. This must be combined with complete portal triad clamping and not with selective left portal clamping to avoid congestion of the middle hepatic vein, which would remain fed by the right lobe.

The right lobe of the liver drains into the right and middle hepatic veins. Therefore, total HVEPC is usually required for right hepatectomies, except in the rare cases of separate implantation of the left and middle hepatic veins, where the left hepatic vein can be left unclamped. The right posterior section (segments 6 and 7) drains into the right hepatic vein and, when present, into the inferior right hepatic vein. In resections restricted to these segments, right posterior partial HVEPC can be applied by clamping the *porta hepatis *or the right portal pedicle and clamping the right hepatic vein(s). In central resections, which include two transection lines, one in the left liver and one in the right liver, alternate sequential partial HVEPC can be applied (5). When the caudate lobe must be removed, it must be completely freed from the IVC by division of all minor hepatic veins.

#### Extreme Resections

Hepatic tumors in the central or posterior segments may involve all of the hepatic veins or may extend to involve the IVC or the hilum, rendering conventional techniques including TVE insufficient. In such cases, the use of unconventional techniques may be required. Despite the availability of innovative surgical techniques that render extensive hepatic resection and concomitant IVC replacement feasible, the surgical death and complication rates associated with this type of surgery remains considerable [31-35]. These techniques include ex-situ or ex-vivo resections and require veno-venous bypass and concomitant hepatic cooling. Derived from liver transplantation, these techniques were reported in several papers in the early 1990s [36-40]. However, poor oncologic results led to their nearly complete abandonment.

Pichlmayr et al., reported a 33% death rate in a series of nine patients who underwent *ex vivo *hepatic resection [41]. In a series of aggressive surgical resection of hepatic metastases involving the IVC, Miyazaki et al., reported a 5-year survival rate of 22% after IVC resection, compared with a 27% survival rate in patients without IVC involvement, however, this was in a small cohort [42]. *Ex vivo *and *in situ *resection of the IVC for hepatic malignancy offers an improved quality of life and a chance for survival in patients with hepatic tumors considered inoperable by standard resection techniques.

The need for *ex-vivo *resection should be rare. The two cases in which we required *ex-vivo *resection had involvement of all three hepatic veins, the inferior vena cava, and portal structures. If only the hepatic veins and IVC are involved, the portal structures can be left intact (though clamped) and the vena cava divided above and below the tumor, allowing the liver to be rotated up to the surface of the operative field. This permits excellent access for reconstruction and reimplantation of the hepatic veins into the vena cava. Hannoun has described a technique where the liver, with portal structures intact, can be flushed via a branch of the portal vein with cold University of Wisconsin solution to extend the ischemic time tolerated by the liver. When complete *ex-vivo *resection is used, or when the liver is flushed with preservation solution, the transection of the liver parenchyma and the reconstruction of vascular structures takes place in a bloodless field and can be done without time constraints. In cases of *ex-vivo *resection, veno-veno bypass may be used to provide hemodynamic stability and portal decompression during the prolonged anhepatic phase of the procedure.

#### Hepatic Tolerance to Vascular Clamping

The main cause of death after hepatectomy is liver failure either due to excessive resection or an underlying liver disease [43,44]. Normal livers tolerate up to one hour of warm ischemia while in diseased livers [i.e., chemotherapy, cholestasis, steatosis, sinusoidal obstruction syndrome (SOS)] ischemia periods must be shorter [45,46]. Transient liver biochemical dysfunction always occurs after hepatic resection with or without clamping. Clamping-induced ischemia may also produce postoperative liver failure, mainly in patients with underlying liver disease or extensive resection, especially when long clamping times have been applied. Therefore, liver function tests must be monitored after liver resection and the surgeon must know how to interpret them. Transaminases correlate well with ischemia and reperfusion injury. Liver failure is best monitored by Prothrombin time or INR and Bilirubin level. Liver ischemia leads to hepatocellular necrosis assessed by an elevation in transaminases, which is directly linked to the duration of clamping [26].

As would be expected, inflow occlusion leads to less severe ischemia than TVE. This can be explained by backflow oxygenation when only simple inflow clamping is utilized. Moderate postoperative elevations of transminases (ALT, AST), rarely exceeding 10-fold normal values, is usually not clinically significant. More than a 20-fold increase of liver enzymes, however, reflects long clamping times and remnant ischemic liver. Postoperative hepatocellular insufficiency including jaundice, encephalopathy and decreased coagulation factors assessed by prothrombin time and INR is a serious complication [47]. Although possible after an extensive hepatectomy on a non-cirrhotic liver, this complication is usually seen in patients with underlying liver disease [48,49]. It is important to note that elevated bilirubin and prothrombin/INR levels may be a more representative and sensitive indication of liver ischemia and liver failure after hepatectomy when compared to transaminitis, which may correlate more with liver regeneration and represent normal laboratory derangements after liver resection [43].

The presence of hepatovenous back-perfusion plays a critical role during the Pringle maneuver. Backflow from the hepatic veins may maintain liver adenosine triphosphate synthesis and, hence, liver viability during the Pringle maneuver [50,51]. The perfusion of liver by hepatovenous backflow, however, is never perfect. In an animal experiment, continuous hepatic inflow interruption for 90 or 120 minutes resulted in severe injury to hepatic cells and liver sinusoids and in multiorgan failure [52,53].

#### Hemodynamic Tolerance

Hemodynamic repercussions of liver vascular clamping have been extensively studied. These are due to portal clamping or to both portal and caval clamping in case of TVE. Hepatic artery clamping alone has almost no significant hemodynamic effect.

Intraoperative hemodynamic assessment during liver resection includes invasive arterial pressure and central venous pressure monitoring. A Swan-Ganz catheter is nowadays rarely required.

The Pringle maneuver is associated with a decrease in cardiac output and an increase in mean blood pressure; the former effect is due to a decreased venous return secondary to portal clamping while the latter effect is linked to arterial vasoconstriction, both mesenteric and systemic. Systemic vasoconstriction is secondary to the decreased venous return. These phenomenona, usually of minimal impact and without significant clinical consequences, do not require any preemptive or therapeutic intervention by the anesthesiologist in the vast majority of cases.

The fluid and pharmacological management by anesthesiologists is of major importance during liver resection and depends on the technique of clamping used by the surgeon, particularly the use of caval clamping or not. In the absence of caval clamping, fluid administration must be as minimal as possible to achieve a low CVP and reduce bleeding during hepatic parenchymal transection. CVP has recently evolved as a major hemodynamic parameter with great influence on operative bleeding in liver surgery.

Intrahepatic pressure depends exclusively on CVP, which is why it must be kept as low as possible during parenchymal transection to minimize bleeding. A CVP of less than 5 mmHg is recommended for all open liver resections without caval clamping [16]. This relative hypovolemia is achieved by reducing fluid administration before and during parenchymal transection. This method, also called "functional" TVE, may however increase, at least theoretically, the risk of gas embolism. Lowering central venous pressure during parenchymal transection mandates a perfect collaboration and trust between an experienced anesthesiology team and the hepatic surgeon. Total inflow occlusion includes portal clamping and therefore generates splanchnic congestion with intestinal edema, which may lead to an increase in postoperative morbidity, especially when intestinal anastomoses have been created.

TVE is associated with major hemodynamic alterations, principally due to caval occlusion, which dramatically reduces the infradiaphragmatic venous return. This leads to major preload reduction with a decrease in blood pressure and cardiac output. Before starting a TVE, it is mandatory to alert the anesthesiologist so that they have sufficient time to provide an adequate vascular load. A five minute-clamping trial is recommended before starting parenchymal transection in order to test the hemodynamic tolerance of the patient. Commonly, arterial blood pressure stabilizes minutes later due to a compensation mechanism based on a 50% to 70% increase in systemic resistances, enhancing the cardiac effort [54]. In addition, mild fluid administration is necessary with or without use of small doses of vasopressors, such as norepinephrine. If hemodynamic stability is achieved at this stage, it can be assumed that it will be maintained for the duration of the transection. When TVE is poorly tolerated despite optimal anesthesiologic management, TVE must be interrupted. Trouble-shooting should begin with assessing whether or not a technical problem with the TVE occurred (i.e. incomplete TVE). In the absence of any technical problems, true hemodynamic insufficiency is most commonly due to low cardiac reserve. In such cases, adrenergic drugs must not be used so that the situation is not worsened. In these situations, the surgeon must choose between pursuing the transection under simple Pringle maneuver or HVEPC and abandoning of the procedure. An ultimate solution may be that of a veno-venous extracorporeal by-pass, either caval or both portal and caval in order to palliate hemodynamic insufficiency. These hazards must not eclipse the fact that TVE is very effective in the majority of cases where it is indicated.

#### Methods to Reduce Ischemic Injury: Intermittent Clamping

The duration of the liver's tolerance to the Pringle maneuver remains a major concern for liver surgeons. Elias et al. reported that an intermittent Pringle maneuver might be used for longer than 120 minutes without major complications, even in diseased livers [55]. Huguet et al., showed that patients with chronic liver diseases had increased postoperative morbidity and mortality rates after 60 minutes of continuous vascular exclusion56. Prolonged hepatic inflow occlusion has been used for up to 60 minutes during hepatectomy without serious complications in selected patients with active chronic liver disease [56].

Clamping periods of 10 to 15 minutes are separated by 5-minute periods of declamping. As compared to continuous inflow clamping, intermittent clamping (IC) does not significantly reduce operative hemorrhage but liver tolerance to ischemia is improved, especially if the parenchyma is abnormal. With IC, cumulative clamping periods of up to 322 minutes in normal liver and 204 minutes in cirrhotic livers have been reported [57]. The aim of IC is to protect liver parenchyma from the deleterious effects of ischemia as well as to reduce prolonged splanchnic venous stasis. In addition, intermittent clamping releases the portal vein in order to minimize bowel congestion and edema. Although intermittent clamping may increase transection time and cause mild oozing during unclamping periods, its advantages outweigh these drawbacks. At the present time, a majority of liver resections deal with diseased livers (i.e., chemotherapy, chronic liver disease) leading most authors to use intermittent clamping when anticipated clamping times are > 30 minutes.

#### Ischemic Preconditioning (IPC)

The discovery of the endogenous cellular protective mechanism known as ischemic preconditioning (IPC) has risen hopes that natural pathways could be activated to help hepatocytes stave off cell death. Short and repeated periods of ischemia inducing unexpected resistance to prolonged ischemia in myocardial cells was first described and defined as IPC by Murry et al. [58]. This mechanism was later shown to occur in other organs including the liver [59-61].

The effects of IPC can be differentiated in 2 phases characterized by different time frames and mechanisms: (1) an ***early phase ***(early preconditioning) that immediately follows the transient hypoxia and lasts 2-3 hours and (2) a ***late phase ***(late preconditioning), which begins 12-24 hours from the transient ischemia and lasts for about 3-4 days. Whereas early preconditioning occurs within minutes and involves the direct modulation of specific cell functions, the late phase requires the simultaneous activation of multiple stress-response genes and the synthesis of several proteins. Despite these differences, both phases of preconditioning can be initiated by the same stimuli and partially share the same intracellular signal pathways. Preconditioning represents a general adaptive phenomenon to sub-lethal stresses capable of increasing cell resistance toward subsequent potential lethal insults. The liver is among the organs in which the preconditioning phenomenon has been shown. A brief (5-10 minutes) interruption of hepatic blood supply in anesthetized rats and mice followed by 10-15 minutes of reperfusion reduces aminotransferase release during a subsequent extended period of ischemia followed by reperfusion. These beneficial effects are also evident in fatty livers, in which preconditioning almost halves the extent of necrosis. Preconditioning procedures have been attempted in pig livers with more variable results [62]. In perfused grafts, IPC effectively prevents reoxygenation injury after 2-hour cold ischemia. However, only minimal protection is evident when preconditioning is applied to anesthetized pigs exposed to warm hepatic ischemia/reperfusion [62].

Preconditioning can also be induced *in vitro *in isolated rat hepatocytes by 10 minutes of incubation under hypoxic conditions followed by 10 minutes of reoxygenation [63]. With both *in vivo *and *in vitro *protocols, the time frame of oxygen deprivation is critical for the induction of preconditioning, because hypoxic periods shorter than 5 minutes or exceeding 15 minutes fail to induce protection. Besides transient ischemia, rat exposure to hyperthermia (42°C for 20 minutes) or mild oxidative stress consequent to the intravenous administration of doxorubicin (1 mg/kg body wt) enhances the hepatic tolerance to reperfusion injury. In these latter conditions, the hepatoprotective effects are evident for up to 48 hours from the treatments, indicating the induction of the late form of preconditioning. Experiments in rodents suggest the possibility of applying preconditioning protocols to improve liver transplantation [64]. The use of both ischemic and heat shock preconditioning before graft harvesting decreases aminotransferase release and sinusoidal endothelial cell death at reperfusion and improves rat survival after orthotopic liver transplantation [63].

The first human trial, described by Clavien in 2000, showed the beneficial effect of a 10-min period of IPC during liver resection [60]. This initial 10-minute period of portal inflow clamping if followed by 10 minutes of reperfusion. Inflow clamping can then be performed continuously or intermittently. In 2003, Clavien showed in a randomized control trial a significant decrease in transaminase levels during the immediate post-operative period in the IPC group [65]. However, no significant effect was observed in morbidity and mortality rates. Other authors confirmed these results for patients having continuous Pringle maneuver, but randomized to either previous IPC or no IPC showed a statistically significant reduction in postoperative ALT levels than in the former group [57,66-68]. One study compared intermittent Pringle maneuver with continuous Pringle maneuver preceded by IPC. Both techniques were shown to be equally safe and effective although intermittent Pringle maneuver caused more bleeding during transection [69].

The impact of liver ischemia on disease progression in humans is unknown. From an oncological point of view, such data would be valuable, especially as data exist from mice models to suggest that the Pringle maneuver promotes the growth of colorectal cancer liver metastases [70,71].

#### Hepatic Cooling

Normothermic ischemia up to 1 hour is well tolerated in normal human livers; however, morbidity rates in patients with chronic hepatic disease or chemotherapy- related modifications are significantly higher [56]. In the majority of liver resections, parenchymal dissection can be completed within 1 hour, but major liver resections with vascular reconstruction may lead to longer periods of normothermic ischemia [56]. If liver ischemia/reperfusion (I/R) injury can be reduced, clamping duration can be safely prolonged, allowing a more prudent, unhurried transection of liver parenchyma and consequent decrease in the risk of technical error, bile leakage and intra- and postoperative hemorrhage. Clearance of blood from the liver without concomitant cooling severely aggravates liver I/R injury [45].

In instances where the projected clamping is prolonged or in cirrhotic livers, some authors have advocated using hypothermia in order to protect the liver [45]. Liver hypothermia is achieved by infusing the liver with a protective solution under TVE. The main portal vein or one of its major branches is cannulated and the outflow is drained by a caval phlebotomy. Once resection is performed and just before declamping, the liver is flushed in order to avoid hyperkalemia. Surface cooling using ice or cold water may be used [40]. Commonly, the procedure is performed under extracorporeal veno-venous, portal and systemic bypass [41].

The use of University of Wisconsin solution was considered for cold *in situ *perfusion of the liver; however, no beneficial effects have been described in the literature as far as short periods of cold ischemia are concerned. Furthermore, the high potassium content of UW solution is a potential hazard when it unintentionally enters the systemic circulation after declamping and must be washed out before reperfusion. Although Ringer-glucose solution in this setting has been associated with encouraging results, further research is warranted to determine the best perfusion solution under these conditions.

## Discussion

Twenty-five percent of Japanese surgeons routinely clamp during liver resections [72]. In a recently published European-wide study, it has been found that vascular clamping is commonly applied during liver resection [73]. One out of 5 surgeons clamp on a routine basis. It is frequent to see that senior surgeons use a standard Pringle maneuver more often, whereas younger surgeons use more selective clamping techniques. Recent reports have indicated that major liver resection can be safely performed without vascular clamping [74,75]. The tendency to withhold from clamping may partly be the result of advances in parenchymal transection, including the use of coagulation devices [76-79].

Pringle maneuver is used when selective clamping is not technically achievable or when tumour location is bilobar or anatomically of difficult access. As compared to the absence of clamping, total inflow clamping significantly may not reduce operative hemorrhage or postoperative complications. During liver surgery for trauma, Pringle maneuver does not need to be used systematically, because simple compression may be as effective in controlling bleeding. Pringle maneuver has almost no systemic hemodynamic repercussions, although some patients with unstable cardiovascular status can present a dangerous arterial pressure decrease, requiring a fluid challenge that increases venous pressure, leading to blood loss from hepatic veins.

Pringle maneuver should be used intermittently and only for non-anatomic resections, or minor resections located close to the terminal part of the hepatic veins (in the inferior parts of the liver) and ideally in patients with normal liver parenchyma. It is also used as an emergency measure in case of bleeding in all types of resections, including laparoscopy. It is also indicated for major hepatic resections provided that the trunk of the major hepatic veins or the inferior vena cava are not involved.

It is important to remember that hepatectomy without clamping is feasible and safe. Living Donor Liver Transplant (LDLT) is the best example. Hepatectomy without clamping does not necessarily mean the absence of vascular control. Refinements in surgical tools and improvements in anesthesiology management allow the surgeon to perform major hepatectomies with an acceptable morbidity rate. Irrespective of this global advancement, part of the morbidity is still related to ischemia-reperfusion injury of the small and diseased remnant liver. Nowadays, modern surgical tools combined with peroperative low CVP may allow a re-evaluation of the value of a systematic clamping policy.

## Appendix 1: Indications for selective clamping

- Unilateral lesions, especially in cases of projected major hepatectomy.

- It is possible to combine and to alternate right and left clamping in cases of lesions located in both hepatic lobes.

- Suprahilar clamping of a sectoral pedicle is more appropriate in cases of bisegmentectomy 5-8 than in cases of right posterior resection (segments 6 and 7).

## Appendix 2: Indications for TVE

1. Whenever there is a risk of a tear of a major intrahepatic vessel, especially hepatic veins in the proximity of the IVC or the IVC itself.

2. When it may be impossible to reduce the central venous:

- Patients with right-sided heart failure

- Pulmonary artery hypertension

- Tricuspid valve insufficiency

## Competing interests

The authors declare that they have no competing interests.

## Authors' contributions

EC participated in the design of the study and its coordinaton, AG participated in the design of the study and its coordination, DC conceived of the study, and participated in its design and coordination. All authors read and approved the final manuscript. Manuscript created in association with iNOELS (Intercontinental Natural Orifice Endo-Laparoscopic Surgery).
